# HIV Protease and Integrase Empirical Substitution Models of Evolution: Protein-Specific Models Outperform Generalist Models

**DOI:** 10.3390/genes13010061

**Published:** 2021-12-27

**Authors:** Roberto Del Amparo, Miguel Arenas

**Affiliations:** 1 Centro de Investigacións Biomédicas (CINBIO), University of Vigo, 36310 Vigo, Spain; rdelamparo@uvigo.es; 2 Department of Biochemistry, Genetics and Immunology, University of Vigo, 36310 Vigo, Spain; 3 Galicia Sur Health Research Institute (IIS Galicia Sur), 36310 Vigo, Spain

**Keywords:** substitution model of protein evolution, protein evolution, phylogenetic reconstruction, viral protease, viral integrase, HIV

## Abstract

Diverse phylogenetic methods require a substitution model of evolution that should mimic, as accurately as possible, the real substitution process. At the protein level, empirical substitution models have traditionally been based on a large number of different proteins from particular taxonomic levels. However, these models assume that all of the proteins of a taxonomic level evolve under the same substitution patterns. We believe that this assumption is highly unrealistic and should be relaxed by considering protein-specific substitution models that account for protein-specific selection processes. In order to test this hypothesis, we inferred and evaluated four new empirical substitution models for the protease and integrase of HIV and other viruses. We found that these models more accurately fit, compared with any of the currently available empirical substitution models, the evolutionary process of these proteins. We conclude that evolutionary inferences from protein sequences are more accurate if they are based on protein-specific substitution models rather than taxonomic-specific (generalist) substitution models. We also present four new empirical substitution models of protein evolution that could be useful for phylogenetic inferences of viral protease and integrase.

## 1. Introduction

Substitution models of molecular evolution are well established in a variety of phylogenetic methods to obtain accurate inferences of past evolutionary processes [1]. At the protein level, substitution models are frequently applied in evolutionary biology to infer phylogenetic trees [2,3], ancestral sequences [4,5] and selection [6,7], among other applications [1,8]. The current substitution models of protein evolution can be classified roughly into two categories. First, there are parametric or structure-based substitution models that consider structural constraints to model selection on the protein folding stability and function [9,10,11,12,13]. These models provided accurate inferences of protein evolution [10,12]; however, although some of them have been implemented in useful evolutionary frameworks [14,15], their mathematical complexity (i.e., most of them account for site-dependent evolution) and large computational requirements prevented (for the moment) their establishment in phylogenetics. The other category includes empirical substitution models of protein evolution [1,8,16]. These substitution models consist of a 20 × 20 matrix of relative rates of change among amino acids (hereafter, an exchangeability matrix) and 20 amino acid frequencies, which are estimated from large protein databases. These models assume that all of the protein sites evolve under the same substitution process, despite the fact that this is often unrealistic (i.e., it is likely that sites located in the catalytic region of an enzyme evolve under different evolutionary patterns compared to sites located at the protein surface due to selection on the protein function [13,15], and these models also ignore the protein folding stability, leading to unrealistically unstable proteins [17]). However, the mathematical simplicity and rapid computation of empirical substitution models favored their establishment in protein phylogenetics, including their implementation in most of the frameworks for phylogenetic tree reconstructions, e.g., [18], and ancestral sequence reconstructions, e.g., [19,20].

Next, most of the empirical substitution models of protein evolution are based on general nuclear (i.e., JTT [21] and WAG [22]) or mitochondrial (i.e., MtMam [16] and mtREV [23]) proteins, and others were developed from proteins of particular taxonomic levels, including viruses like the human immunodeficiency virus (HIV) [24], influenza virus [25], dengue virus [26] and flavivirus [27], among others. Still, the currently available set of empirical substitution models of protein evolution is very limited, with less than 100 substitution models [1]. Next, it is known that the accuracy of phylogenetic inferences depends on the applied substitution model [28,29,30,31,32]; consequently, the selection of the best-fitting substitution model of evolution currently constitutes a fundamental step in phylogenetics [33]. The limited number of currently available empirical substitution models of protein evolution means that, for a particular dataset of protein sequences, one could not find an appropriate substitution model. For example, using a framework for substitution model selection, the authors of [34] found that the best-fitting empirical substitution model for datasets from proteobacteria and archaea was a model inferred from retroviral Pol proteins, which is likely to improperly describe the evolutionary processes of the cited datasets. Therefore, there is a need for more empirical substitution models of protein evolution, at least while realistic structure-based substitution models are not yet established in phylogenetics. Regarding this concern, as noted previously, most of currently available empirical substitution models of protein evolution are largely generalist (i.e., a single substitution model is based on all of the different proteins existing in a taxonomic level). Regarding this concern, we believe that empirical substitution models that are specific for protein families could more accurately mimic the evolution of a protein dataset belonging to the underlying protein family. For example, at present, phylogenetic inferences from a dataset of HIV protease (PR) or integrase (IN) sequences can be performed under the HIVw or HIVb substitution models [24], which are the only currently available empirical substitution models based on HIV proteins. However, these two empirical substitution models are based on all of the different proteins present in this virus, and thus we consider them to be generalist. As a consequence, we believe that these models can be highly unrealistic when modeling a dataset of a specific HIV protein (i.e., PR or IN). This intuitive reasoning motivated us to investigate whether a protein-specific empirical substitution model of evolution could outperform the currently available set of generalist empirical substitution models of evolution that are commonly used in phylogenetics. In order to test this hypothesis, and also to provide new empirical substitution models that can be useful for certain viral phylogenetic inferences, we developed and evaluated four novel protein-specific empirical substitution models of evolution. In particular, we developed two models for viral PR (one for the HIV PR and another one for the PR of multiple viruses; hereafter, HIVpr and VIRpr, respectively) and two models for viral IN (one for the HIV IN and another one for the IN of multiple viruses; hereafter, HIVin and VIRin, respectively). Next, we evaluated the fitting of these models with other models (including HIVb and HIVw) using independent test data.

## 2. Materials and Methods

**Figure 1:**
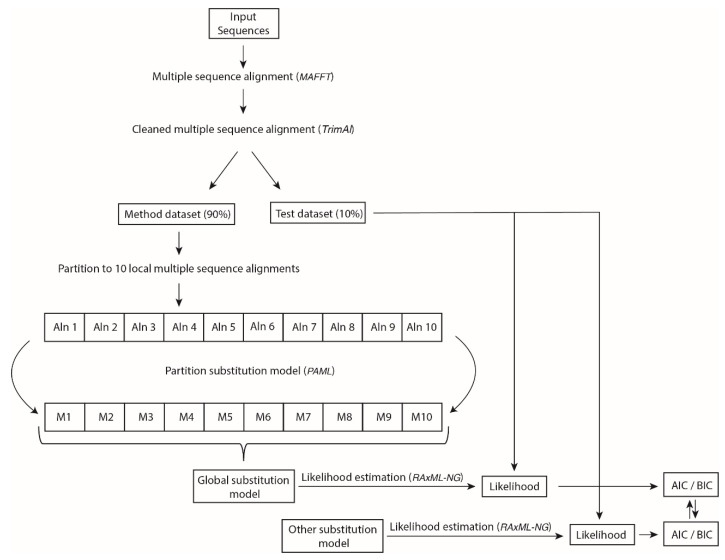
Pipeline for the inference and evaluation of the empirical substitution models of PR and IN evolution. The input protein sequences were aligned and cleaned (removing duplicate sequences and uninformative sites). Next, the resulting multiple-sequence alignment (MSA) was split into two datasets: a method dataset (for the inference of the substitution model, including most of the sequences) and a test dataset (for the evaluation of the substitution model). Indeed, the method dataset was split into 10 local method datasets (due to computational limitations), and we inferred a local (partition) substitution model for each one. The resulting local substitution models were averaged to obtain a global substitution model. Finally, we calculated the AIC and BIC scores for the global substitution model and other currently available empirical substitution models in order to evaluate them, considering the likelihood of every model with the test dataset.

In this section, we describe the data collection, the development of the empirical substitution models of PR and IN evolution, and the evaluation of the developed models by likelihood-based comparisons with currently available empirical substitution models. All of these methodological steps are illustrated in Figure 1.

### 2.1. Study Data of HIV-1 and General Virus Protease and Integrase

We collected all of the protein sequences of HIV PR and IN available in GenBank to develop the substitution models of HIV PR and IN, respectively. We only considered sequences with a length similar to the natural length of HIV PR (99 amino acids) and IN (163 amino acids) in order to avoid uninformative sequences with multiple indels. We obtained a total of 55,000 and 23,000 sequences for HIV PR and IN, respectively. Next, for each dataset, we obtained a multiple-sequence alignment (MSA) using MAFFT [35]. The resulting MSAs were further refined by removing sequences with multiple gaps (we only allowed sequences with less than 30% gaps) with TrimAl [36], following previous studies, e.g., [25,37]. The final MSAs included 16,900 and 14,764 sequences with lengths of 99 and 163 amino acids for the HIV PR and IN, respectively. Concerning the development of substitution models based on PR and IN from multiple viruses, we collected sequences of viral PR and IN from the PFAM database (codes PF00077 and PF00665 for the PR and IN, respectively). We also applied the previously indicated filtering to obtain the final MSAs, which included a total of 1605 and 34,282 sequences for the viral PR and IN, respectively.

### 2.2. Inference of Novel Empirical Substitution Models for HIV and General Virus Protease and Integrase

We split every dataset in two datasets: (i) the method dataset, which includes 90% of the sequences, and was used to infer the substitution model, and (ii) the test dataset, which includes 10% of the sequences, and was used to evaluate the developed substitution model. The inference of an empirical substitution model requires a large number of sequences [38], and therefore we incorporated most of them into this group. However, we note that 10% of the sequences provided to the test datasets include a large number of sequences (1700 and 1500 for HIV PR and IN, respectively; 144 and 3400 for general viral PR and IN, respectively). Actually, we benefited from the large number of sequences that are available for these proteins. Note that they have been frequently sequenced due to their relevant role as common antiretroviral drug targets [39,40,41,42]. Next, we found that the large number of sequences present in some method datasets (in particular, those with more than 10,000 sequences, which are 3 of the 4 method datasets) caused computational limitations that forced us to split them into 10 partitions with the same size (Figure 1). For each partitioned method dataset, we inferred an empirical substitution model (in particular, the exchangeability matrix and amino acid frequencies) under the maximum-likelihood (ML) method implemented in *PAML* [19], using an ML phylogenetic tree previously reconstructed with *RAxML-NG* [18] under the best-fitting substitution model selected by *ProtTest* [43]. We allowed *PAML* to internally optimize the model and phylogenetic tree according to the input data [19]. Using the ML method implemented in *PAML*, we obtained 10 local exchangeability matrices and sets of amino acid frequencies that we applied to calculate (by their average) the global exchangeability matrix and amino acid frequencies (Figure 1).

### 2.3. Evaluation of the Novel Empirical Substitution Models of HIV-1 and General Virus Protease and Integrase

We evaluated the inferred empirical substitution models using the test datasets. First, we applied *ProtTest* to every test dataset in order to identify the best-fitting empirical substitution model among the currently available empirical substitution models. Next, we obtained the likelihood and the Akaike Information Criterion (AIC) [44] and Bayesian Information Criterion (BIC) [45] scores of the fitting of every substitution model (including the corresponding empirical substitution model developed in this study and the top 5 of the currently available empirical substitution models that best fit with the studied test dataset and were selected by *ProtTest*) with the test dataset using *RAxML-NG* (Figure 1). Additionally, we applied a statistical *t*-test to compare the AIC and BIC scores obtained from the empirical substitution models developed in this study with the scores obtained from other currently available empirical substitution models (the top 5 best-fitting empirical substitution models among the set of currently available empirical substitution models) with the test datasets.

## 3. Results and Discussion

### 3.1. Novel Empirical Substitution Models for HIV and General Virus Protease and Integrase

We developed protein-specific empirical substitution models of evolution for the viral PR (one for the HIV PR and another one for the PR of multiple viruses; HIVpr and VIRpr, respectively) and for the viral IN (one for the HIV IN and another one for the IN of multiple viruses; HIVin and VIRin, respectively). These four new empirical substitution models of protein evolution are presented in Tables S1–S4 (Supplementary Material). The developed substitution models are based on symmetric exchangeability matrices (*Q*), which despite potentially being more unrealistic than asymmetric exchangeability matrices, are well-established in phylogenetics due to the more simple calculation of the probability matrix (*P*) for a given period of time (*t*), *P*(*t*) = *exp*(*Qt*). Therefore, the development of symmetric exchangeability matrices allows a wider implementation of the models in phylogenetic frameworks.

**Figure 2:**
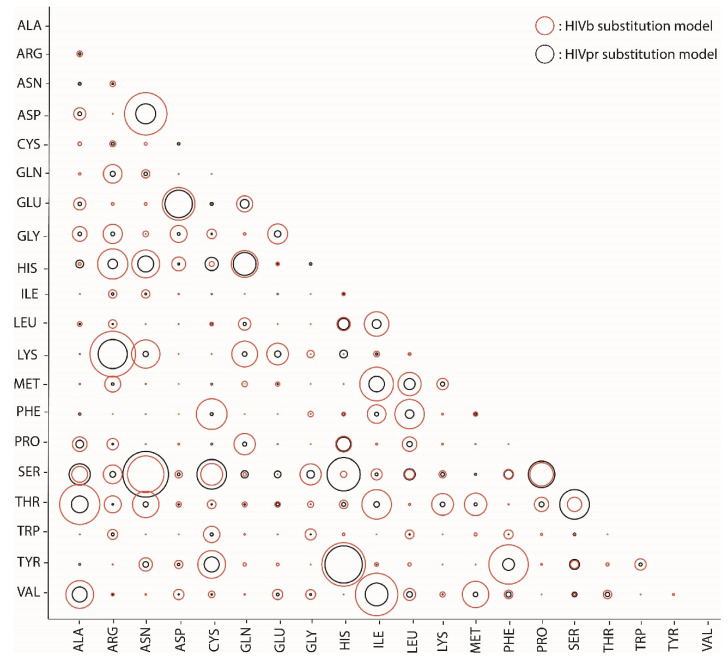
Comparison of HIVpr and HIVb empirical substitution models concerning their relative substitution rates. The plot displays the exchangeability matrix of the relative substitution rates among amino acids for the HIVpr (developed in this study, black circles) and HIVb (the best-fitting substitution model in the set of currently available substitution models, red circles) empirical substitution models of evolution. This plot provides an illustrative comparison between the cited models; the specific parameter values of the HIVpr substitution model are presented in Table S1.

We found that, among the currently available empirical substitution models (excluding the models developed in the present study), the HIVb substitution model produced the best fitting with all of the test datasets, except for the viral IN test dataset, for which the selected model was WAG. Next, we present qualitative comparisons (quantitative comparisons are shown in the next section) between the substitution models developed in this study and the currently available best-fitting substitution models (Figure 2 and Figures S1–S3, Supplementary Material). In general, we found that the HIVpr and VIRpr substitution models decreased (compared with the HIVb model) most of relative rates of change among the amino acids considering the PR function, which suggests a higher specificity. For example, the PR presents a catalytic aspartic acid (Asp-25) that is usually conserved due to selection to maintain the protein function [46,47]. Consequently, if it is not conserved, it could only change to glutamic acid (also a potent acidic nucleophile) in order to conserve the physicochemical properties and functionality of the catalytic region. In agreement with the consequences of this selection pressure, the HIVpr and VIRpr substitution models presented a lower substitution rate from aspartic acid to any other amino acid (compared with HIVb), except for its substitution to glutamic acid that presents physicochemical properties similar to aspartic acid (Figure 2 and Figure S1). We only found some amino acid changes where the HIVpr or VIRpr substitution models displayed a higher relative substitution rate than the HIVb substitution model, such as the substitutions serine/threonine and serine/asparagine (in HIVpr), which involve amino acids with similar physicochemical properties. Less-intuitive cases involved the substitutions serine/histidine (in HIVpr) or lysine/tyrosine (in VIRpr), which imply a physicochemical change between polar and basic amino acids, and can occur at the protein surface involved in the protein solubility [46,47]. Concerning comparisons between the HIVin and HIVb substitution models, and between the VIRin and WAG substitution models (note that HIVb and WAG were selected as the best-fitting substitution models among the currently available set of empirical substitution models, excluding the models developed in this study), we found again that the HIVin and VIRin models present more restrictive relative rates of change for the aspartic and glutamic acids than the selected models (HIVb and WAG) (Figures S3 and S4). Again, note that the main catalytic sites of the integrase are aspartic and glutamic acids (Asp-64, Asp-116 and Glu-152) [48,49]. Comparing HIVin and HIVb, or VIRin and WAG, we also observed a few amino acid changes with an increase in their relative rate of change, such as isoleucine/methionine, isoleucine/leucine, leucine/methionine and serine/threonine (in HIVin) or cysteine/tyrosine (in VIRin), where the amino acids involved presented similar physicochemical properties, agreeing with selection pressure for the maintenance of the protein function [40,50,51].

### 3.2. Likelihood-Based Comparisons Indicate That the Novel Empirical Substitution Models Outperform the Currently Available Empirical Substitution Models

**Figure 3:**
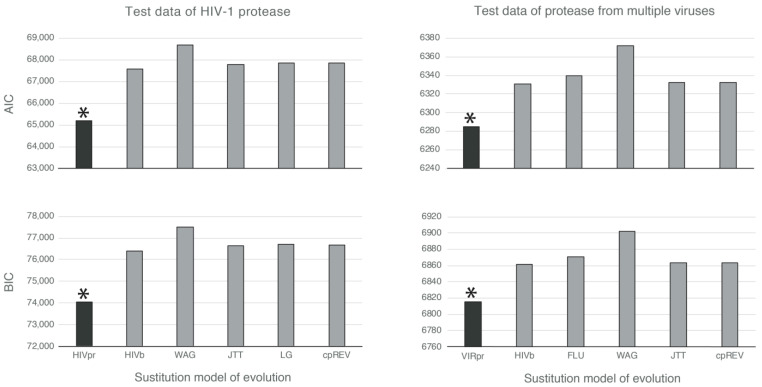
Likelihood-based evaluation of the HIVpr, VIRpr and currently available best-fitting substitution models. For the HIV PR (left plots) and viral PR (right plots) test datasets, the plots show the AIC (top plots) and BIC (bottom plots) scores obtained with the HIVpr and VIRpr substitution models inferred in this study and the top five currently available best-fitting substitution models with the corresponding test dataset. In all of the cases, the models developed in this study produced AIC and BIC scores (black bars) significantly lower than the currently available best-fitting substitution models (*p*-values = 0.00013 and 0.00014 for HIVpr and VIRpr, respectively and illustrated with * in the plots).

For every test dataset, we found that the novel substitution model that we inferred for every corresponding protein family outperforms the currently available best-fitting substitution models in terms of likelihood. In particular, we found that the currently available best-fitting substitution models (excluding the substitution models developed in this study) for the test datasets of HIV PR, viral PR, HIV IN and viral IN were HIVb, HIVb, HIVb and WAG, respectively. Next, all of the models developed in this study (HIVpr, VIRpr, HIVin and VIRin) provided lower AIC and BIC scores than the cited currently available best-fitting substitution models, and also than the top five currently available best-fitting substitution models (*p*-values < 0.05; Figure 3 and Figure S4; Supplementary Material). These results indicate that phylogenetic analyses of viral PR and IN are more accurate if they are based on a substitution model of evolution developed from the corresponding studied protein family (such as the substitution models developed in the present study) than if they are based on a generalist substitution model such as those which are currently available.

## 4. Conclusions

Substitution models of protein evolution are required for the most accurate phylogenetic reconstruction methods. However, the currently available set of empirical substitution models is highly limited, and mostly includes generalist models that are based on huge protein groups (i.e., nuclear or mitochondrial proteins) or on all of the proteins of a particular taxonomic level; they thus lack specificity when studying a particular protein family. Here, we show that there is a need for protein-specific empirical substitution models of evolution because they can provide accurate likelihood-based phylogenetic inferences, and we demonstrate this with the development and evaluation of four new empirical substitution models that mimic the substitution process of the PR and IN of HIV and other viruses. Of course, the accurate inference of protein-specific empirical substitution models of evolution requires a large number of protein sequences, but we believe that with the current exponential increase of protein sequences being deposited in databases, this limitation will be greatly reduced with time. Altogether, we conclude that, in order to obtain more accurate phylogenetic inferences for protein families, protein-specific empirical substitution models should be developed and applied. Indeed, we believe that the new empirical substitution models that we present in this study could be useful for evolutionary studies of viral PR and IN, which are some of the main targets of current antiretroviral drug-based treatments.

## Data Availability

The input data and the developed empirical substitution models are available at the Zenodo repository from the URL https://doi.org/10.5281/zenodo.5763867 (accessed on October 2021) [52].

## Supplementary Materials

The following supporting information can be downloaded at https://www.mdpi.com/article/10.3390/genes13010061/s1, Table S1: Empirical substitution model of HIV PR evolution (HIVpr); Table S2: Empirical substitution model of viral PR evolution (VIRpr); Table S3: Empirical substitution model of HIV IN evolution (HIVin); Table S4: Empirical substitution model of viral IN evolution (VIRin); Figure S1. Comparison of VIRpr and HIVb empirical substitution models concerning their relative substitution rates among amino acids; Figure S2: Comparison of HIVin and HIVb empirical substitution models concerning their relative substitution rates among amino acids; Figure S3: Comparison of VIRin and WAG empirical substitution models concerning their relative substitution rates among amino acids; Figure S4: Likelihood-based evaluation of the HIVin, VIRin and the currently available best-fitting substitution models.
