# Supplementary material for: HIV Protease and Integrase Empirical Substitution Models of Evolution: Protein-Specific Models Outperform Generalist Models
Source: Genes (Basel). 2021 Dec 27;13(1):61. doi: 10.3390/genes13010061 (PMC8774313; doi:10.3390/genes13010061)
Supplement: Supplementary file 1 [file genes-13-00061-s001.zip › genes-1525398-supplementary.pdf]

Supplementary Material

HIV protease and integrase empirical substitution models of evolution: Protein-specific models outperform generalist models

The supplementary material includes Tables S1-S4 and Figures S1-S4.

Supplementary Tables

6.32
10.55 3.35
19.19 0.57 528.74
17.2 10.24 4.57 9.03
0.44 33.95 16.1 0.93 0.48
16.84 2.54 2.12 995.35 11.86 102.71
17.71 19.07 0.26 10.38 2.68 0.11 53.78
79.92 119.8 337.27 5.51 231.83 690.54 1.36 8.08
0.57 7.3 4.23 0.29 1.92 0.49 2.37 0.07 1.33
2.18 1.91 0.07 0.07 7.3 13.06 0.15 0.12 163.34 104.13
1.31 1121.9 37.04 0.16 0.07 27.74 49.82 0.2 80.21 5.72 0.23
1.02 8.77 1.3 0.07 4.03 0.07 1.26 0.36 0.07 318.75 161.89 23.06
6.76 0.07 0.53 0.07 11.29 0.07 0.07 0.35 9.56 21.11 96.13 0.07 5.85
78.87 3.49 0.34 1.9 4.05 22.17 0.41 0.08 258.01 0.22 37.55 0.07 1.24 0.07
591.8 44.14 2744.87 10.21 1158.6 69.86 59.15 78.82 1457.3 12.1 164.73 18.26 6.23 101.04 929.6
368.98 3.51 35.4 2.06 2.01 2.56 13.35 0.45 20.63 40.82 2.16 28.55 12.67 0.67 32.8 1162.32
0.58 10.78 0.07 0.07 11.48 0.42 0.67 1.26 0.07 0.07 2.65 0.07 1.98 0.62 0.22 8.74 0.2
4.38 0.07 43.1 8.55 298.72 2.15 2.5 0.32 1841.14 0.57 1.02 0.07 0.07 182.38 0.07 133.54 0.07 12.13
294.95 1.81 0.14 1.92 3.11 0.29 10.41 4.81 0.07 684.17 41.79 0.85 28.45 35.39 0.07 31.2 26.2 0.07 0.07

0.031 0.038 0.038 0.045 0.02 0.059 0.039 0.13 0.007 0.127 0.111 0.066 0.02 0.02 0.063 0.004 0.081 0.02 0.01 0.07

Table S1. Empirical substitution model of HIV PR evolution (*HIVpr*). The table shows the inferred exchangeability matrix (relative rates of change among amino acids) and amino acid frequencies from HIV PR sequences, in the following order of amino acids: A R N D C Q E G H I L K M F P S T W Y V. The model can also be obtained from Zenodo (link in the article) as a text file. A graphical representation of the matrix is shown in Figure 2.

10.47
1.63 68.53
17.06 8.7 282
90.41 116.84 14.07 6.18
14.55 208.89 43.25 2.69 14.32
62.9 54.39 159.37 119.83 0.04 270.59
33.54 65 28.59 21.14 18.87 3.12 46.44
0.04 644.32 416.64 36.02 159.35 663.26 122.66 0.04
2.46 13.34 24.89 3.96 12.6 0.04 8.71 0.44 32.14
7.58 22.93 4.03 0.45 7.09 13.28 1.98 0.5 2.16 163.54
15.38 453.19 377.64 0.04 0.04 275.52 482.96 3.71 57.56 11.29 19.36
71.29 55.95 7.96 11.13 131.67 44.96 38.99 5.33 0.04 257.71 257.61 46.49
54.47 0.04 0.04 5.15 153.31 0.04 0.04 2.21 54.12 87.31 162.59 45.13 162.39
69.47 83.4 77.62 33.07 0.04 63.06 66.7 5.89 99.55 3.05 66.23 142.24 0.04 0.04
178.87 68.02 293.42 11.81 124.62 68.41 31.97 39.34 21.08 4.38 22.46 56.7 0.04 76.33 245.07
103 46.99 67.85 8.18 24.75 57.94 39.82 4.77 31.65 42.09 0.04 136.25 334.89 0.04 81.39 254.75
23.7 180.82 0.04 0.04 56 19.48 0.04 31.38 0.04 32.53 97.7 30.56 0.04 245.96 9.79 76.84 10.35
0.04 27.98 441.55 92.7 615.32 2.59 0.04 4 2006.42 27.74 32.34 1054.62 494.12 884.51 24.48 207.58 40.22 313.08
130.01 23.12 2.84 1.4 110.95 5.62 3.39 7.73 13.29 357.9 74.69 19.43 319.54 104.77 3.21 3.73 27.45 0.04 18.66

0.06 0.04 0.027 0.073 0.022 0.04 0.034 0.125 0.014 0.088 0.13 0.03 0.017 0.02 0.019 0.058 0.089 0.007 0.005 0.101

Table S2. Empirical substitution model of viral PR evolution (*VIRpr*). The table shows the inferred exchangeability matrix (relative rates of change among amino acids) and amino acid frequencies from PR sequences of diverse viruses, in the following order of amino acids: A R N D C Q E G H I L K M F P S T W Y V. The model can also be obtained from Zenodo (link in the article) as a text file. A graphical representation of the matrix is shown in Figure S1.

7.41
28.95 14.85
2.52 0.19 331.94
3.7 39.01 0.19 0.19
5.35 319.03 49.58 0.19 0.19
38.63 9.49 7.04 560.05 1.85 89.7
44.18 124.67 27.25 40.96 7.81 4.3 94.46
2.41 78.73 232.14 14.78 3.87 290.94 4.72 0.73
0.19 5.64 4.09 0.19 0.19 0.19 4.17 0.34 0.19
1.59 9.07 3.69 0.19 0.19 35.32 7.06 0.19 41.05 1517.45
2.02 1909.53 204.8 0.19 0.19 171.01 37.41 1.06 1.62 1.06 1.87
0.19 183.14 0.19 0.19 4.82 0.19 0.19 1.69 0.19 2096.61 1089.28 6.51
0.19 3.75 1.04 1.24 14.27 0.49 0.19 0.51 1.42 5.23 246.33 1.88 0.19
99.6 20.94 0.19 0.19 0.19 61.04 0.19 1.96 15.41 1.98 47.66 0.83 4.43 0.19
205.33 386.64 338.32 0.92 358.85 16.21 1.67 97.25 18.49 2.42 6.71 4.7 6.2 13.57 939.76
733.82 80.17 432.09 0.19 3.48 31.66 2.74 3.59 2.33 222.18 2.86 106.43 351.38 0.19 79.04 844.88
0.19 14.03 0.19 1.13 3.14 0.19 0.19 2.06 0.19 0.19 1.43 0.19 0.19 0.19 0.19 0.19 0.19
0.19 74.45 4.45 5.61 193.56 0.19 0.19 0.73 112.12 3.31 6.22 1.87 0.19 316.07 0.19 4.15 3.11 2.61
128.14 12.46 0.19 3.18 5.83 0.19 4.1 5.87 1.47 1861.48 236.91 0.43 187.85 12.96 0.19 4.28 128.52 0.19 0.19

0.0922 0.0263 0.0338 0.0361 0.0186 0.0632 0.0686 0.096 0.0371 0.1011 0.0465 0.0688 0.0183 0.0307 0.0316 0.0431 0.0566 0.0246 0.0248 0.082

Table S3. Empirical substitution model of HIV IN evolution (*HIVin*). The table shows the inferred exchangeability matrix (relative rates of change among amino acids) and amino acid frequencies from HIV IN sequences, in the following order of amino acids: A R N D C Q E G H I L K M F P S T W Y V. The model can also be obtained from Zenodo (link in the article) as a text file. A graphical representation of the matrix is shown in Figure S2.

34.96
37.58 73.4
38.22 8.7 391.25
190.48 199.12 54.93 19.68
82.12 349.92 198.69 25.68 24.22
144.2 27.67 125.76 303.64 6.36 416.7
109.91 79.35 149.32 79.42 61.12 28.99 78.42
49.95 320.66 496.35 50.82 95.95 491.03 50.99 44.74
10.95 16.38 25.39 1.76 63.39 9.74 4.89 1.76 11.77
20.64 27.61 7.6 1.37 117.39 66.92 5.28 2.53 47.52 333.9
52.88 547.39 362.67 13.52 16.32 355.5 283.4 35.19 70.24 19.34 15.8
76.2 60.85 39.46 3.53 123.12 88.32 20.1 5.96 54.15 588.65 670.13 76.41
12.73 2.59 5.34 1.52 139.72 2.01 2.19 2.98 47.66 68.68 238.28 1.86 131.5
108.29 24.33 5.26 6.51 38.37 41.04 11.32 7.94 36.28 8.62 48.34 18.45 12.78 4.23
295.27 54.5 301.19 29.94 196.61 61.61 40.96 115.25 51.99 12.19 35.75 47.45 15.93 37.48 87.17
294.71 57.6 179.9 19.84 106.19 76.97 47.1 9.43 47.45 155.23 21.95 132.74 277.47 4.46 31.09 388.5
7.73 95.29 3.79 0.77 135.81 37.11 10.3 26.28 50.97 23.83 57.81 5.69 73.77 211.02 3.21 16.11 15.99
14.95 8.34 66.15 37.98 607.4 23.56 5.98 4.07 545.51 14.26 16.02 7.38 47.73 760.11 3.15 26.44 9.47 310.61
232.77 11.04 5.22 7.59 154.14 19.34 22.63 11.55 9.89 903.95 177.38 6.62 324.95 48.4 21.13 7.77 148.34 33.5 10.29

0.068 0.062 0.032 0.074 0.017 0.036 0.048 0.056 0.026 0.063 0.078 0.051 0.02 0.048 0.048 0.077 0.069 0.016 0.035 0.074

Table S4. Empirical substitution model of viral IN evolution (*VIRin*). The table shows the inferred exchangeability matrix (relative rates of change among amino acids) and amino acid frequencies from IN sequences of diverse viruses, in the following order of amino acids: A R N D C Q E G H I L K M F P S T W Y V. The model can also be obtained from Zenodo (link in the article) as a text file. A graphical representation of the matrix is shown in Figure S3.

Supplementary Figures

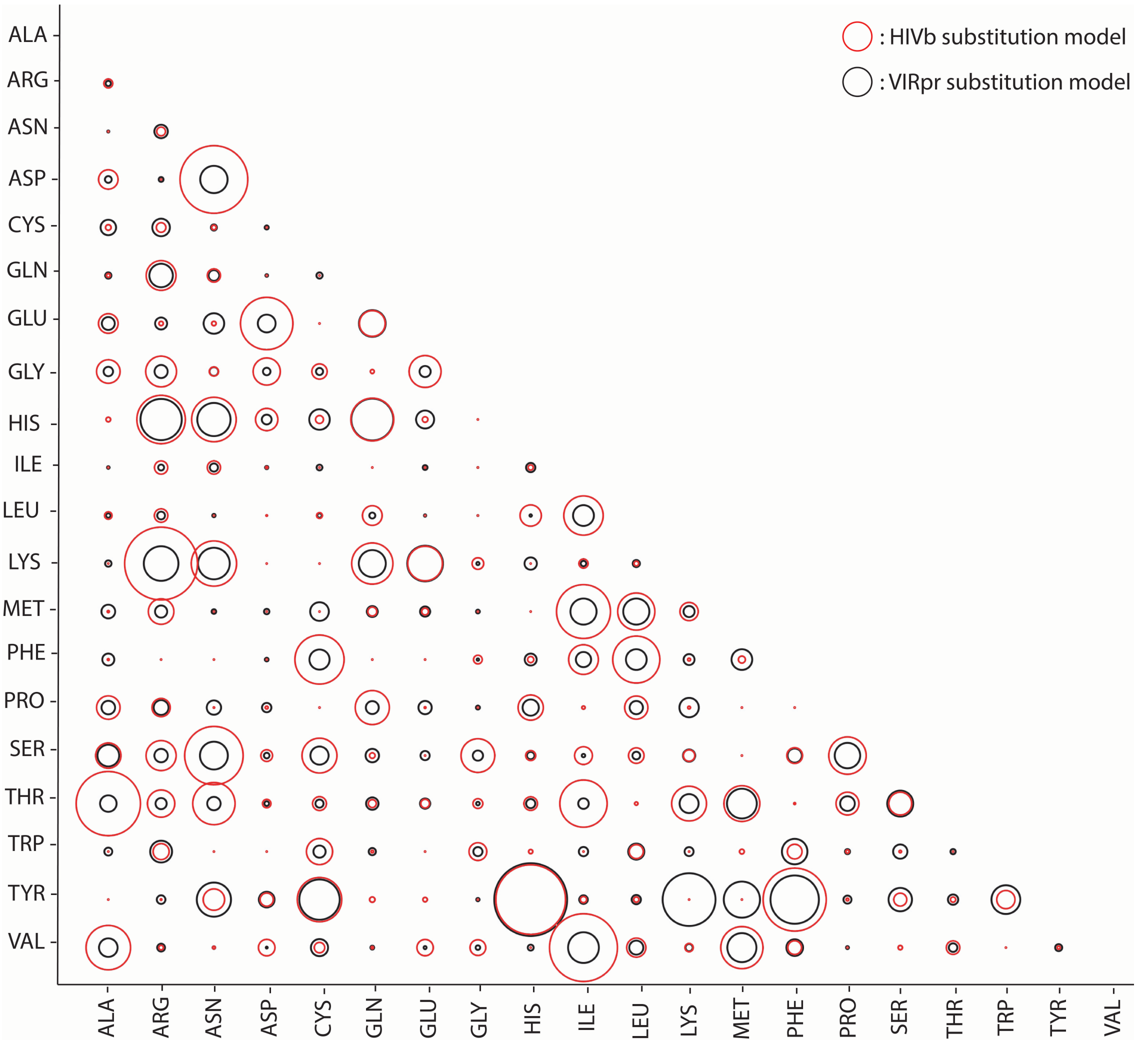

Figure S1. Comparison of VIRpr and HIVb empirical substitution models concerning their relative substitution rates among amino acids. The plot displays the exchangeability matrix of relative substitution rates among amino acids for the VIRpr (developed in this study; black circles) and HIVb (best fitting substitution model from the currently available set of substitution models; red circles) empirical substitution models of evolution. This plot provides an illustrative comparison between the cited models, the specific parameter values of the VIRpr substitution model are presented in Table S2.

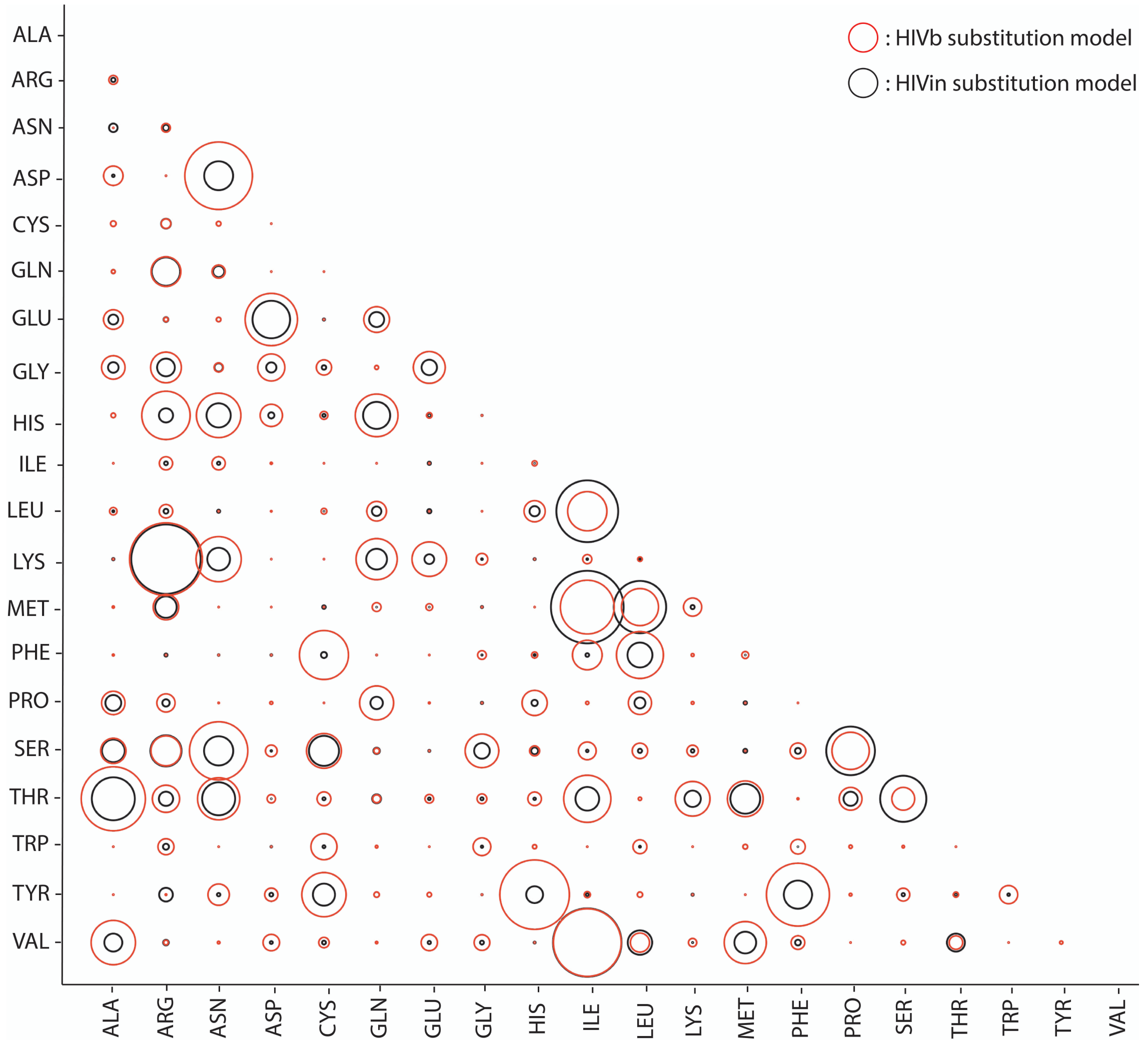

Figure S2. Comparison of HIVin and HIVb empirical substitution models concerning their relative substitution rates among amino acids. The plot displays the exchangeability matrix of relative substitution rates among amino acids for the HIVin (developed in this study; black circles) and HIVb (best fitting substitution model from the currently available set of substitution models; red circles) empirical substitution models of evolution. This plot provides an illustrative comparison between the cited models, the specific parameter values of the HIVin substitution model are presented in Table S3.

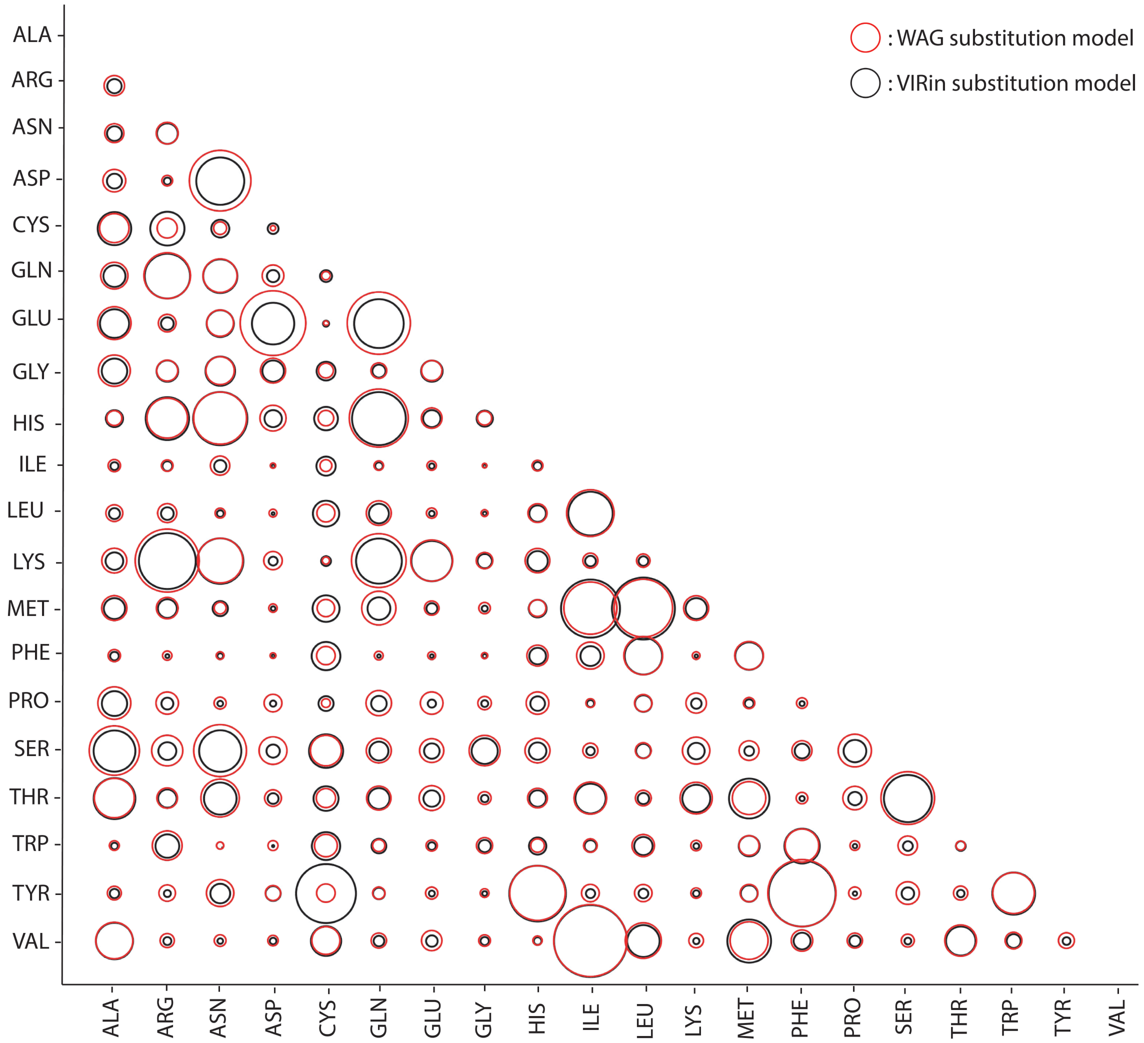

Figure S3. Comparison of VIRin and WAG empirical substitution models concerning their relative substitution rates among amino acids. The plot displays the exchangeability matrix of relative substitution rates among amino acids for the VIRin (developed in this study; black circles) and WAG (best-fitting substitution model from the currently available set of substitution models; red circles) empirical substitution models of evolution. This plot provides an illustrative comparison between the cited models, the specific parameter values of the VIRin substitution model are presented in Table S4.

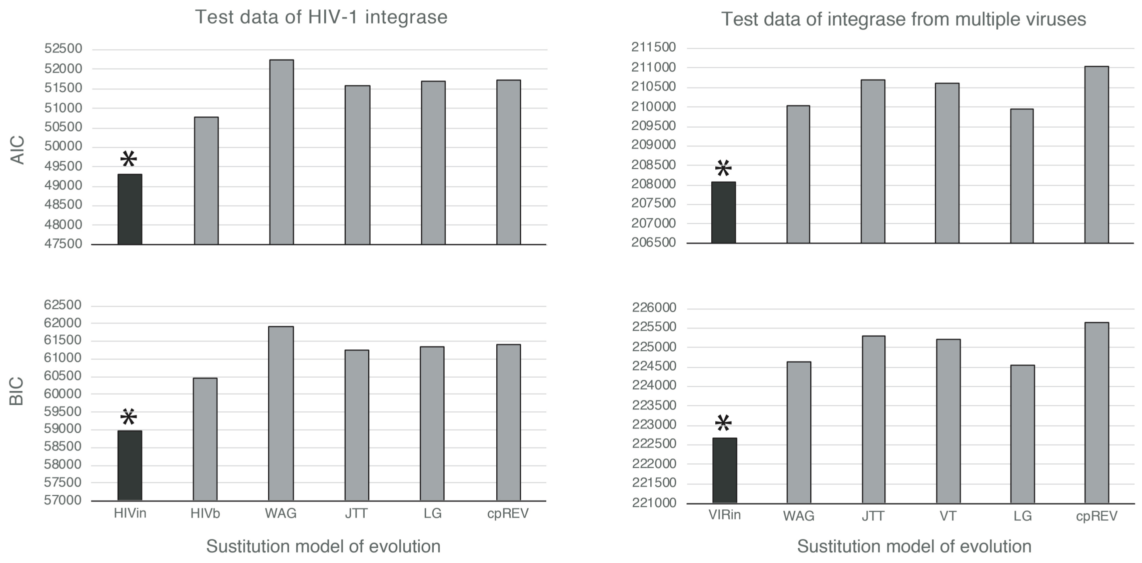

Figure S4. Likelihood-based evaluation of the HIVin, VIRin and the currently available best-fitting substitution models. For the HIV IN (left plots) and viral IN (right plots) test datasets, the plots show the AIC (above plots) and BIC (below plots) scores obtained with the HIVin and VIRin substitution models inferred in this study and the top 5 of currently available best-fitting substitution models with the corresponding test dataset. In all the cases, the models developed in this study produced AIC and BIC scores (black bars) significantly lower than the currently available best-fitting substitution models (p-value = 0.00062 and 0.00033 for HIVin and VIRin, respectively).
